# Tissue distribution and cell tropism of *Brucella canis* in naturally infected canine foetuses and neonates

**DOI:** 10.1038/s41598-018-25651-x

**Published:** 2018-05-08

**Authors:** Tayse Domingues de Souza, Tatiane Furtado de Carvalho, Juliana Pinto da Silva Mol, João Vítor Menezes Lopes, Monique Ferreira Silva, Tatiane Alves da Paixão, Renato Lima Santos

**Affiliations:** 1 Departamento de Clínica e Cirurgia Veterinárias, Escola de Veterinária, Universidade Federal de Minas Gerais, Belo Horizonte, Minas Gerais Brazil; 2 Curso de Medicina Veterinária, Universidade Vila Velha, Vila Velha, ES Brazil; 3 Departamento de Patologia Geral, Instituto de Ciências Biológicas, Universidade Federal de Minas Gerais, Belo Horizonte, Minas Gerais Brazil

## Abstract

*Brucella canis* infection is an underdiagnosed zoonotic disease. Knowledge about perinatal brucellosis in dogs is extremely limited, although foetuses and neonates are under risk of infection due to vertical transmission. In this study, immunohistochemistry was used to determine tissue distribution and cell tropism of *B*. *canis* in canine foetuses and neonates. Diagnosis of *B*. *canis* in tissues of naturally infected pups was based on PCR and sequencing of amplicons, bacterial isolation, and immunohistochemistry, whose specificity was confirmed by laser capture microdissection. PCR positivity among 200 puppies was 21%, and nine isolates of *B*. *canis* were obtained. Tissues from 13 PCR-positive puppies (4 stillborn and 9 neonates) presented widespread immunolabeling. Stomach, intestines, kidney, nervous system, and umbilicus were positive in all animals tested. Other frequently infected organs included the liver (92%), lungs (85%), lymph nodes (69%), and spleen (62%). Immunolabeled coccobacilli occurred mostly in macrophages, but they were also observed in erythrocytes, epithelial cells of gastrointestinal mucosa, renal tubules, epidermis, adipocytes, choroid plexus, ependyma, neuroblasts, blood vessels endothelium, muscle cells, and in the intestinal lumen. These results largely expand our knowledge about perinatal brucellosis in the dog, clearly demonstrating a pantropic distribution of *B*. *canis* in naturally infected foetuses and neonates.

## Introduction

*Brucella canis* is a zoonotic coccobacillus described for the first time as a cause of canine abortion in 1966^1^. Since then, *B*. *canis* has been recognized as the etiologic agent of reproductive disorders and, less frequently, of discospondylitis and ocular infection in dogs^2^. Although *B*. *canis* is recognized as an important bacterial zoonosis, human brucellosis is mainly related to *B*. *melitensis*, *B*. *abortus*, and *B*. *suis*, while brucellosis due to *B*. *canis* infection in humans has historically been considered less relevant^3^. Zoonotic infection may occur mainly in humans in close contact with bitches that aborted. However, several cases of human brucellosis due to *B*. *canis* infection occurred in owners of dogs with no history of abortion, and these patients presented unspecific symptoms when compared to those of patients infected with other *Brucella* spedies, which may lead to the lack of clinical suspicion and diagnostic failure^4–8^. Although these case reports have not been characterized by a labour-related transmission, human infection with *B*. *canis* is also considered an occupational underdiagnosed disease^9^.

*B*. *canis* differs from other *Brucella* species in its pathogenicity and preferential host, affecting mainly dogs, and, occasionally, humans and wild canids^2,3^. Antigenically, *B*. *canis* is classified as a rough *Brucella* due to its outer membrane lipopolysaccharide that characterizes a rough surface on colonies grown for more than 48 hours^10^. Rough lipopolysaccharide, also naturally expressed in *B*. *ovis*, is associated with impaired intracellular survival *in vitro*, and induction of lower levels of cytokine expression in human monocytes^11,12^. Furthermore, *B*. *canis* does not cross-react in traditional serological test antigens from smooth species, namely *B*. *melitensis*, *B*. *abortus*, and *B*. *suis*^13^. The lack of cross-reactivity between smooth and rough *Brucella* antigens in serology contributes to diagnostic failure and under diagnosis of *B*. *canis* human infections^5,9^.

*B*. *canis* induces less inflammation and more insidious lesions when compared to other *Brucella* spp.^14^, but its ability to persist in the host, disseminate and perpetuate in dog population, is demonstrated by its prevalence in many countries^15,16^, and the difficulty for clearing the pathogen from infected dogs^17^ and eradicating it in kennels^18–20^. Diagnosis of *B*. *canis* infection remains a challenge due to frequent false negative results in direct and indirect diagnostic methods employed to detect the infection in adult dogs and humans^6,21,22^.

Despite the relevance of canine brucellosis as a reproductive disease that is vertically transmitted to the offspring^2,19,23^, and the high canine perinatal mortality rates of unknown cause in breeding kennels^24,25^, canine perinatal brucellosis remains neglected worldwide. One of the reasons for this situation is that *B*. *canis* infection is suspected mostly in cases of abortion, but not in cases of perinatal mortality^1,10,15^.

Cellular and tissue tropism of *B*. *canis* in its natural preferential host remains poorly understood. It has been assessed based on isolation and histopathology^10,26^, but not through *in situ* localization of the bacteria. In canine foetal brucellosis, bronchopneumonia, myocarditis, and renal haemorrhage have been described in an experimental study^26^, while neonatal canine brucellosis remains to be described. Therefore, canine brucellosis is a disease that has been neglected both in man and in the dog, possibly due to its stealthy behaviour, difficulty for diagnosis, and limited knowledge about *B*. *canis* infection, treatment, and eradication. A better understanding of canine brucellosis in foetuses and neonates will contribute for improving diagnosis and prevention of this disease in dogs and humans. The aim of this study was to describe cell and tissue tropism of *B*. *canis* in naturally infected stillborn and neonatal dogs, which generated a novel perspective on *B*. *canis* pathogenesis, with implications regarding the persistence of the pathogen and the risk of zoonotic transmission.

## Results

### PCR, sequencing, and *Brucella* sp. isolation

A total of 42 puppies out of the 200 (21%) that were tested, from 16 kennels, were PCR-positive. Amplicon sequences had 100% identity with *Brucella* spp. (GenBank access number: M20404.1).

*B*. *canis* was isolated from tissues of six out the 10 puppies subjected to bacterial isolation, totalling 42 tissue samples. From these six puppies, nine isolates were obtained from placenta (n = 2), spleen (n = 2), kidney (n = 2), heart (n = 2), and lung (n = 1). Isolates and their origins are listed in Table 1.

**Table 1:** *Brucella*-positive puppies, their breed, kennel, age, bacterial isolates origin, and positivity by immunohistochemistry in organs and tissues.

Puppy	Breed	Kennel	Puppy age at death	Isol.	GIT	Heart	Lung	Spleen	Liver	Kidney	Urinary bladder	LN	Adipose tissue	Blood	Genital	Umbilical Cord	CNS	Eye
1 (127)	German Spitz	A	Stillborn	NE	+++	++	++	+	+++	+	+	−	−	++	+	+++	NA	NA
2 (143)	German Spitz	B	Stillborn	Placenta	+++	++	+++	+	+	+	++	+	+	++	++	+++	NA	NA
3 (30)	Border Collie	C	Stillborn	NE	++	+++	−	+++	++	++	+	−	++	+++	−	+	++	NA
4 (33)	Brazilian Mastiff	D	Stillborn	Placenta Kidney	+	+++	−	−	+	+++	+	−	+	−	++	+++	NA	++
5 (53)	Miniature Pinscher	E	1 day	Lung Heart	++	+	++	++	+	+	+	+	+	−	+	+++	NA	NA
6 (51)	Miniature Pinscher	E	2 days	Kidney Spleen	++	−	++	+	+++	++	+	+	−	−	++	+++	++	NA
7 (16)	Miniature Poodle	E	2 days	NE	+++	+	++	−	+	+	−	−	+	++	−	+	++	−
8 (156)	Rottweiler	F	2 days	NE	+++	+	+	+	++	+	+	+	+	+	NA	++	NA	NA
9 (185)	English Bulldog	G	2 days	NE	+++	+	+++	−	+	++	++	+	−	−	NA	++	NA	NA
10 (90)	Miniature Pinscher	E	3 days	NE	+++	+	+++	+++	+++	++	+++	+++	++	++	+++	+++	++	++
11 (50)	German Spitz	H	4 days	NE	+	+	+++	−	++	++	+	++	+	++	+	NA	NA	NA
12 (111)	Yorkshire Terrier	I	10 days	Spleen	++	++	++	+	−	++	+	+	++	−	−	NA	NA	NA
13 (112)	German Spitz	A	13 days	Heart	+++	−	+++	−	+	++	NA	+	++	−	++	NA	NA	NA
% Positive					100	85	85	62	92	100	92	69	77	54	73	100	100	67

Abbreviations: GIT: Gastrointestinal tract; LN: Lymph node; CNS: Central nervous system; NE: Negative; NA: Not available to be tested. + = only one positive mark in ten fields (400X) or more; ++ = at least one positive mark in every two fields; +++ = more than two positive marks in more than ten fields.

### Pantropic distribution of *Brucella canis* in foetal and neonatal canine tissues

**Figure 1:**
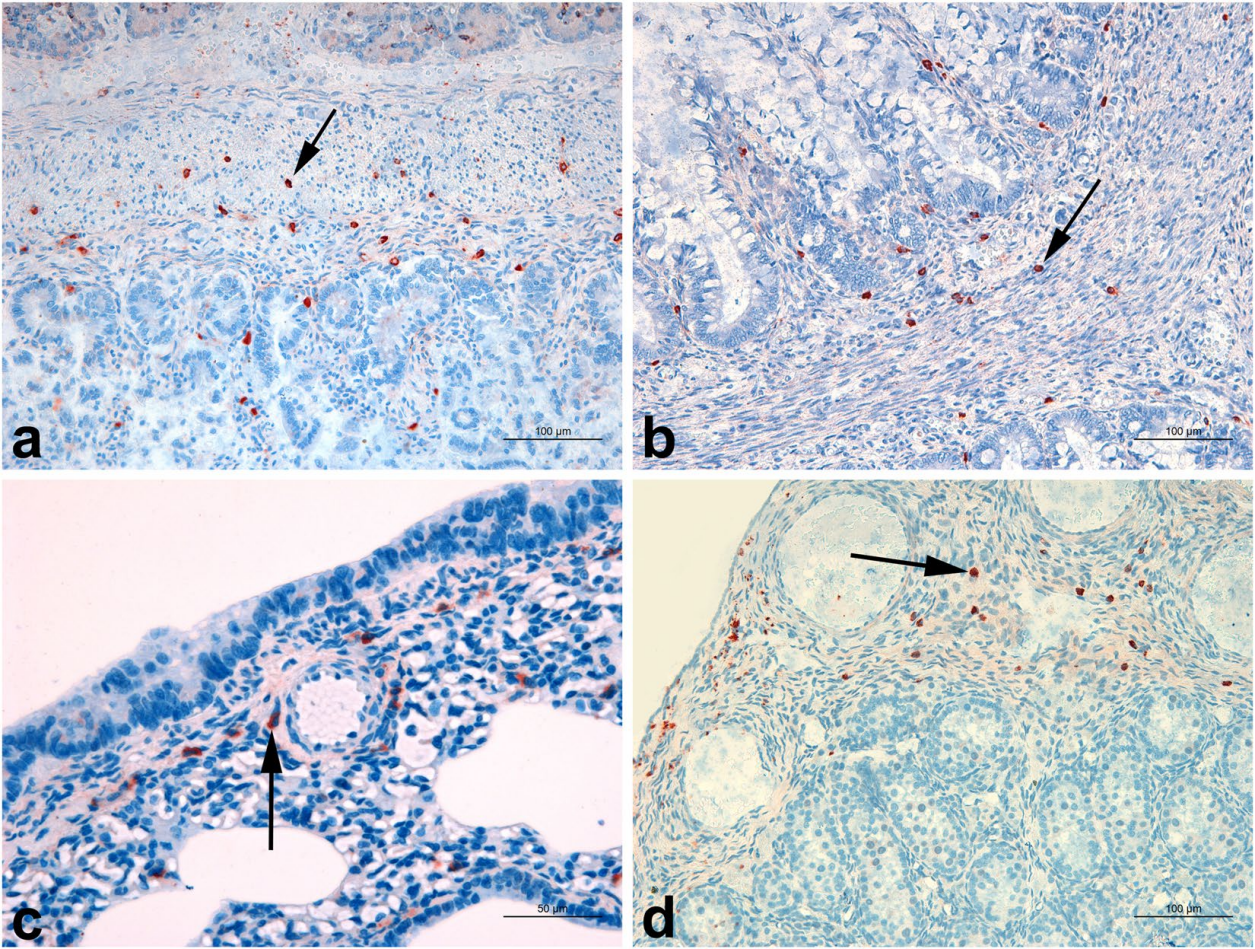
Immunohistochemistry for detection of *Brucella* sp. in naturally infected canine stillborn and neonates. (**a**) Stillborn. Duodenum, with several diffusely distributed immunolabeled macrophages in the lamina propria and submucosa (arrow). Bar = 100 μm. (**b**) Stillborn. Cecum, macrophages containing intracytoplasmic immunolabeled coccobacilli in lamina propria and muscle layer (arrow). Bar = 100 μm. (**c**) Neonate, 10-day-old. Lung, with several interstitial immunostained macrophages around an arteriole and bronchioles (arrow). Bar = 50 μm. (**d**) Neonate, 13-day-old. Testicle with several immunostained macrophages in the albuginea (arrow). Bar = 100 μm.

Immunolabeled coccobacilli were widely distributed in several organs and tissues from all 13 *Brucella* PCR-positive puppies (Table 1). Organs with the highest frequency of positivity (100%) included the stomach, intestine, kidney, and umbilicus, which contained numerous intracytoplasmic immunolabeled coccobacilli located mostly within macrophages (Fig. 1). Macrophages were also positive in the hepatic parenchyma as well as in portal spaces and around central lobular veins (12/13), in lungs (11/13), and heart (11/13). Reproductive organs, including the gonads, uterus and prostate, although pre-pubertal and in a very young age, were frequently positive (8/11), with immunolabeling mostly in macrophages, in the albuginea (Fig. 1), prostate, myometrium, uterine broad ligament. Spleen and lymph nodes, positive in 62–69% of the puppies, most of the time presented low intensity (−or +, 10/13; − or +11/13), respectively. In these tissues, positive macrophages often had perivascular localization (Fig. 1), and infiltrated the wall of arteries (pulmonary, umbilical, coronary, and hepatic).

**Figure 2:**
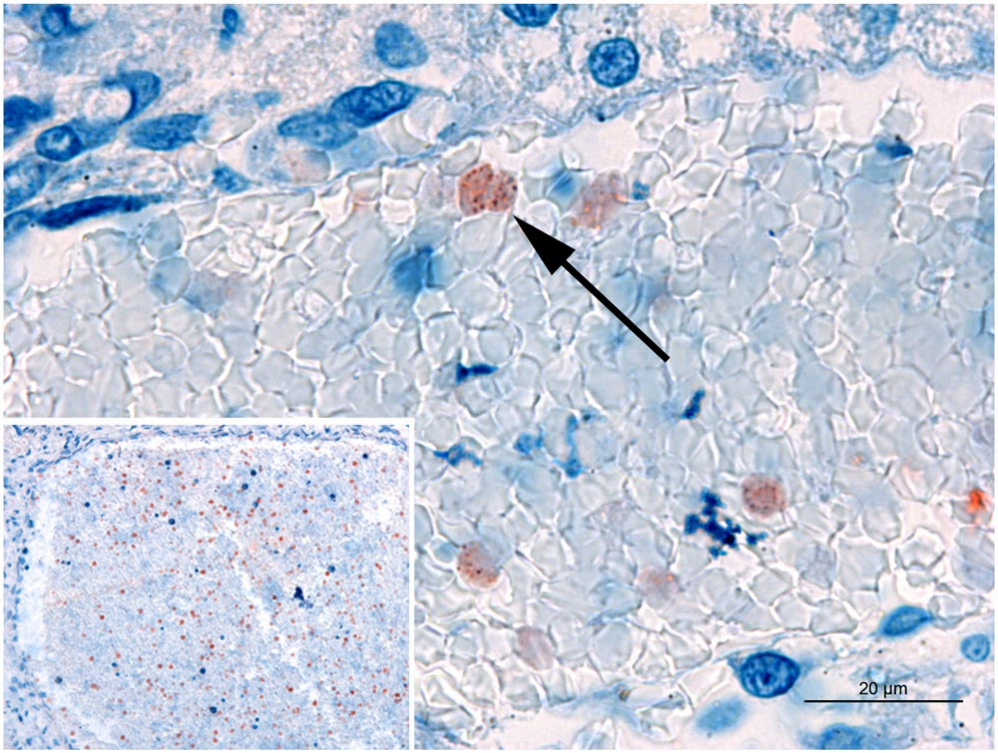
Immunohistochemistry for detection of *Brucella* sp. in stillborn dogs. Erythrocytes in a blood vessel in the kidney with granular immunostaining compatible with intraerythrocytic *B*. *canis* (arrow). Inset: Blood in a cardiac chamber with moderate number of immunostained erythrocytes. Bar = 20 μm.

**Figure 3:**
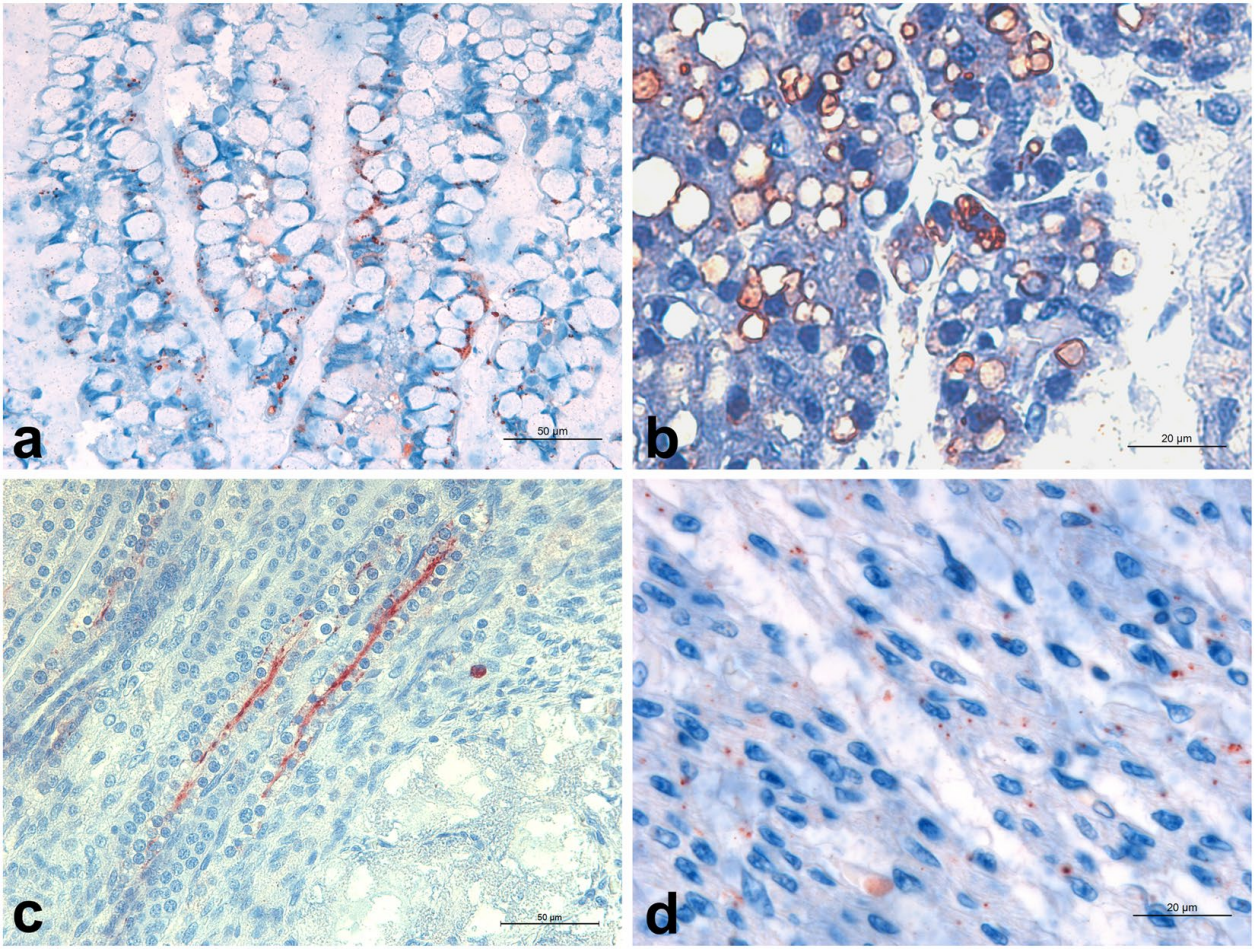
Immunohistochemistry for detection of *Brucella* sp. in canine foetuses and neonates. (**a**) Neonate, 2-day-old. Ileum with numerous intracellular coccobacilli in goblet cells of the villus epithelium. Bar = 50 μm. (**b**) Stillborn. Immunostained adipocytes in the gastro-splenic ligament, with a granular pattern adjacent to lipid vacuoles. Bar = 20 μm. (**c**) Neonate, 2-day-old. Kidney with granular immunostaining in tubular epithelial cells and in tubular lumen. Bar = 50 μm. (**d**) Stillborn. Myocardium with intracellular granular immunostaining in myocardial cells. Bar = 20 μm.

There was also positive coccoid staining in intravascular erythrocytes (7/13) in different organs, including the lungs, genital organs, kidneys, liver, heart chambers, and cerebrum (Fig. 2). In addition to macrophages and erythrocytes, positivity was also observed in other cell types in different organs, as in superficial epithelium of gastric mucosa, in enterocytes, and in goblet cells in the small and large intestines (Fig. 3). Meconium was positive in 75% of stillborn puppies while only 33% of neonates had immunolabeled coccobacilli in the intestinal luminal content, with a lower intensity of immunolabeling (+, 3/9). The gastrointestinal mucosa was intensely positive in all puppies, although immunolabeled coccobacilli were observed mostly intracytoplasmically in macrophages diffusely distributed in the lamina propria, submucosa, muscular layer, mesentery and gastro-splenic ligament, in stillborn and neonates. Only two puppies had low intensity of immunolabeling in stomach and intestine: one premature stillborn and one four-day-old premature neonate who did not nurse in the bitch and was fed artificial milk formula by orogastric tube due to palatoschisis. In five puppies, immature adipocytes in the mesenteric fat contained immunolabeled coccobacilli in the cytoplasm (Fig. 3).

In five puppies, three stillborn and two neonates, *Brucella* was observed intracytoplasmically in the epithelial cells of proximal and distal convoluted tubules, Henle loops, and collecting tubules (Fig. 3). Immunolabeled coccobacilli occurred also intracytoplasmically in adipocytes in perirenal fat (++, 2/13).

**Figure 4:**
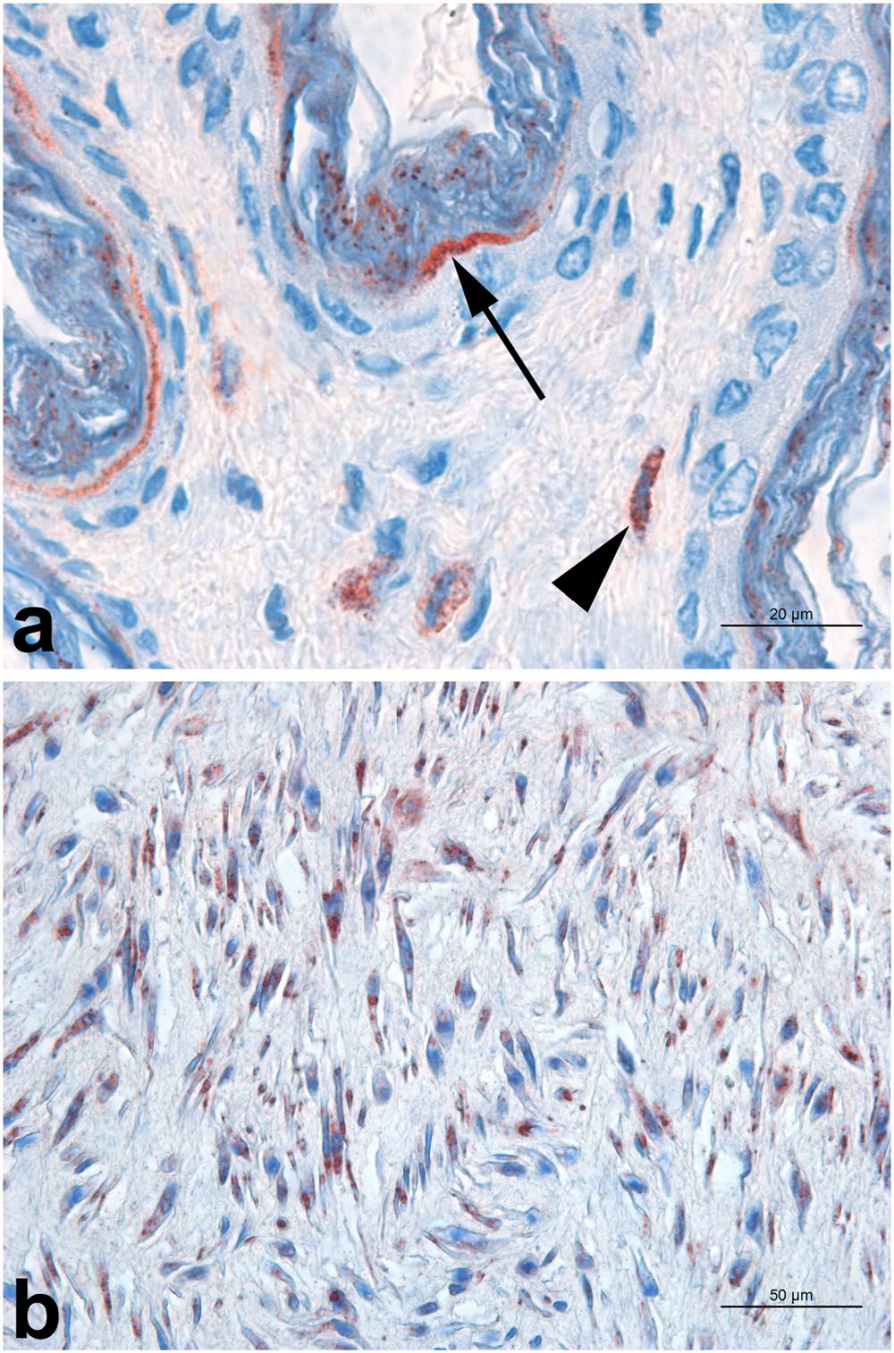
Immunohistochemistry for detection of *Brucella* sp. in the umbilical cord of a stillborn dog. (**a**) Skin adjacent to the umbilicus with immunolabeled coccobacilli in *stratum corneum* (arrow) and in macrophages in the dermis (arrow heads). Bar = 20 μm. (**b**) Arterial wall in the umbilical cord with intracellular immunostained coccobacilli in smooth muscle cells. Bar = 50 μm.

Occasionally, intracytoplasmic immunolabeled coccobacilli were present in smooth muscle cells in umbilical, pulmonary and coronary arteries, where few or no macrophages and neutrophils were present (Fig. 4). The same finding was observed in myocardium cells, with variable number of coccobacilli intracytoplasmically in the absence of inflammation or associated with rare interstitial macrophages and neutrophils (Fig. 3).

In the umbilical cord, immunostained coccobacilli occurred on the epidermis in the *stratum corneum* (Fig. 4) in stillborn and neonates (+ to +++), and also intracytoplasmically in the apocrine gland epithelial cells (++) in only one sample, and in adipocytes in subcutaneous fat (+, 3/10), besides the positivity in macrophages and in arterial smooth muscle cells already mentioned above.

**Figure 5:**
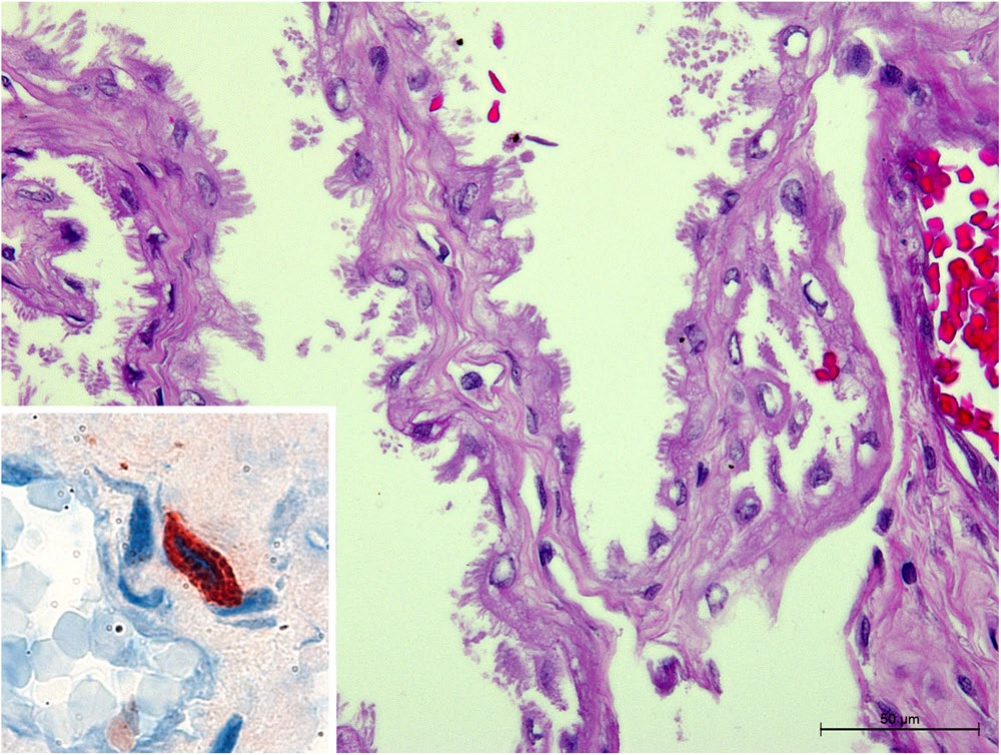
Immunohistochemistry for detection of *Brucella* sp. in the placenta. Section of the foetal placenta infected with *Brucella canis*, with absence of inflammatory reaction. Haematoxylin and eosin. Bar = 50 μm. Inset: intracellular immunostaining in a cell located in the mesenchyme with morphology compatible with a macrophage.

There were only two placentas available for this study, and they were both positive by immunohistochemistry, with intracellularly immunolabeled *B*. *canis* predominantly in macrophages located in the foetal mesenchyme, and occasionally within trophoblasts (Fig. 5). Although, immunolabeling was observed in macrophages, there was no evidence of inflammatory reaction in haematoxylin and eosin-stained sections (Fig. 5), and those cells were interpreted as resident macrophages of the placenta.

**Figure 6:**
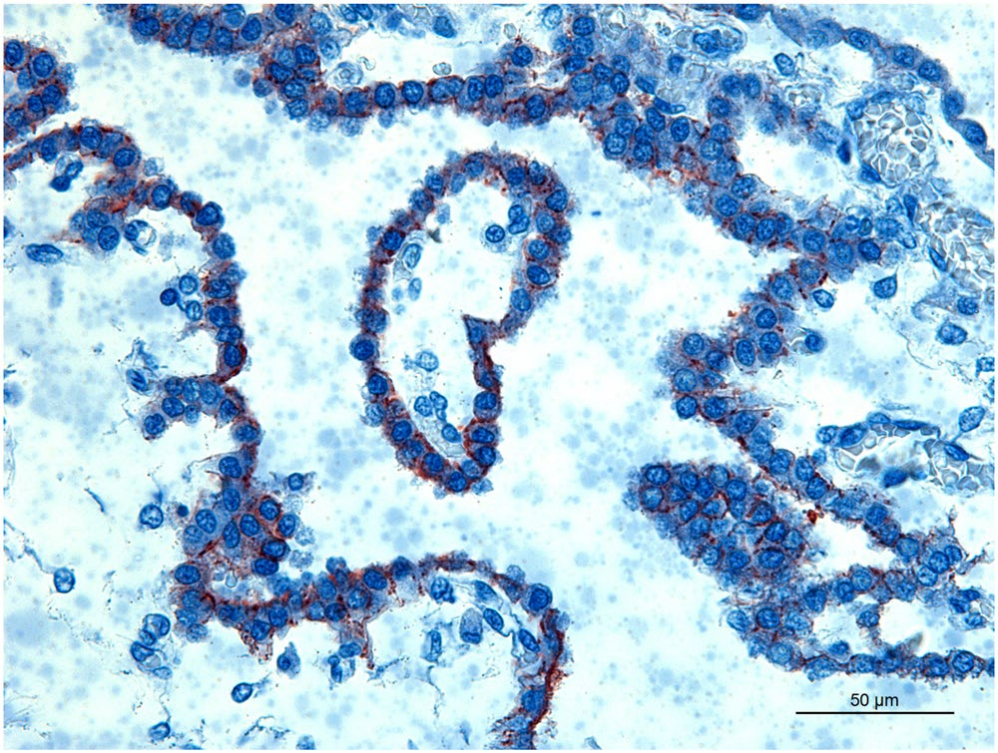
Immunohistochemistry for detection of *Brucella* sp. in the choroid plexus of a stillborn dog. Choroid plexus with intracytoplasmic immunostaining with a granular pattern in cells of the choroid plexus. Bar = 50 μm.

In the nervous tissue, there was positive staining intracytoplasmically in the choroid plexus (Fig. 6) (++ to +++, 3/4), ependyma (+ to ++, 3/4), neuroblasts (+ to +++, 2/4), and in macrophages in the meninges (+, 1/3). In the eye, there was a mild immunostaining in the cornea and retina (+, 2/3), while in the dermal surface of the eyelids there were numerous macrophages containing positive coccobacilli (++, 2/3).

Extracellular immunolabeled coccobacilli occurred rarely, in small numbers, in the lumen of renal tubules, intestinal content and epicellular on choroid plexus cells.

Considering the originality of these findings, immunolabeled epithelial cells of renal tubules were microdissected by using a laser capture microdissection apparatus. DNA extracted from these immunolabeled microdissected immunolabeled renal tubular epithelia was PCR-positive for *Brucella* sp., further confirming the specificity of immunolabeling.

## Discussion

This study largely expanded our knowledge regarding the tissue and cell tropism of *B*. *canis* in canine foetuses and neonates, clearly demonstrating a previously unknown pantropic distribution of this pathogen in several organs and tissues. This is the first thorough description of *B*. *canis* tissue distribution and cell tropism in its preferential host, and therefore this study contributed to a better understanding of canine brucellosis.

Abundant immunohistochemistry positivity in the gastrointestinal mucosa and in intestinal contents suggested that the oral route of infection may play an important role in canine perinatal brucellosis, possibly through ingestion of bacteria in suspension in the amniotic fluid. Carmichael and Kenney^26^ hypothesized that foetal infection could occur through the oral route, based on their findings of high bacterial loads in the amniotic fluid and mild inflammation observed in the digestive system of canine foetuses. Furthermore, high positivity in the gastrointestinal mucosa was also observed in this study in one neonate at 12 days after birth, which may be due to transmammary transmission. Importantly, *B*. *abortus* endocytosis by M cells in the Peyer’s patches had been described after oral experimental infection of calves^27^ and in mice^28^, but the pathogen has not been observed within enterocytes and goblet cells. While the gastrointestinal mucosa and mesentery were highly positive in all puppies in this study, mesenteric lymph nodes were negative in most of the puppies, which is possibly due to the early stages of infection in these cases.

The ability of *B*. *canis* to infect renal tubular epithelial cells strongly suggested a previously unsuspected route of excretion of the organism. Transmission of *B*. *canis* has been attributed to prolonged contact with urine of an infected dog, but it was assumed that the organism originated from prostatic secretions of adult males^29^. Although the prostate gland in puppies was also positive, immunolabeled coccobacilli were located in interstitial macrophages, while in renal tubules they were in epithelial cells and in the lumen, indicating a source for urine contamination in the kidney itself. Zoonotic transmission to humans in close contact with young puppies has been documented^5,8^, and based on our findings, it may have occurred due to shedding of the organism in the urine, since in these cases abortion or vaginal secretions that are considered the conventional means of zoonotic transmission were absent^23^.

This study has demonstrated for first time that *B*. *canis* is associated with erythrocytes in naturally infected canine foetuses and neonates. Canine brucellosis is recognized as a disease with intermittent cell-associated bacteremia, presumably intracytoplasmically in leucocytes^2,23,26^. *B*. *melitensis* is capable of infecting erythrocytes of experimentally infected mice, in which bacterial cells were demonstrated either epi- or intracellularly in erythrocytes^30^. However, association of *Brucella* spp. with erythrocytes has not been previously demonstrated in naturally infected animals. Distribution in tissues and frequency of positivity in erythrocytes was variable, a finding that is consistent with the intermittent detection of *B*. *canis* in the blood of infected dogs^21^.

*B*. *canis* infection in myocardial cells and arterial smooth muscle cells had not been documented before. It may explain the frequent finding of myocarditis and arteritis associated with this disease^26^. In humans, arterial aneurysm has been diagnosed in a teenager infected with *B*. *canis*^4^, what may have occurred due to the direct infection of the smooth muscle cells.

This study also demonstrated that *B*. *canis* are often associated with cells of the choroid plexus. Importantly, neurobrucellosis is a highly relevant clinical presentation in different host species^31^. *B*. *abortus* has been described infecting neuroblasts and glial cells^32^, but, in dogs, encephalic neurological signs are usually not attributed to *B*. *canis* infection. Based on our findings, research on neurobrucellosis in dogs should be encouraged, and perhaps this agent should be included among the differentials in canine neurological patients.

Interestingly, immunostaining was observed in genital organs of canine foetuses and neonates. *B*. *canis* tropism to the genital tract in males has been well documented in adult dogs^33^, but it has not been documented in prepubertal animals. The early infection in epididymis and testicles may compromise irreversibly the reproductive potential of the dogs. Interestingly, in the uterus, positivity was observed in myometrium, and seldom in endometrium. *Brucella* spp. are known to have tropism for the placenta^34^, although there are clearly differences in terms of clinical and pathological manifestations according to combination of *Brucella* and host species^35^. Interestingly, the two placentas available for this study had *B*. *canis* infection as demonstrated by bacterial isolation and immunohistochemistry, but no inflammatory reaction was observed histologically. This contrasts with the severe necrotizing and neutrophilic placentitis observed in other species, particularly in *B*. *abortus*-infected pregnant cows^34^. However, these observations require additional studies since only two samples are not sufficient for a definitive conclusion.

Ocular lesions have been reported in adult dogs infected with *B*. *canis*^17^. Therefore, immunolabeling in the cornea and retina in neonates is not unexpected, but it indicated that infection and ocular disease may occur in perinatal brucellosis, even before the opening of the eyelid.

*B*. *canis* infection of adipocytes has not been previously described, although adipocytes are permissive cells to bacterial infection. *Mycobacterium tuberculosis* in human adipocytes has been demonstrated by IHQ and *in situ* PCR, especially in adipose tissue adjacent to kidneys, lymph nodes, stomach, heart and skin^36^, the same distribution observed for *B*. *canis* in this study. Adipose tissue may represent a site for persistence of *Brucella* infection in dogs, a localization that is suitable to evade treatment and immune response, as is the case for *M*. *tuberculosis* in humans^37^. In adipocytes, the endoplasmic reticulum is localized adjacent to the lipid vacuole^38^, the same location where the immunohistochemistry positive coccobacilli were visualized in this study. Furthermore, adipocytes in canine foetuses and neonates are immature and undergoing differentiation. In a previous *in vitro* study, pre-adipocytes expressed unfolded proteins during the differentiation process^39^ a response also observed during *Brucella* intracellular replication^40^, which suggests that differentiating adipocytes may represent a viable niche for *Brucella* replication. The presence of *B*. *canis* in mature adipocytes remains to be investigated.

Apart from the infected macrophages, immunolabeling was not associated with other inflammatory cells or significant inflammation in tissues, what seems to be a feature of *B*. *canis*, since naturally infected dogs do not develop signs of sepsis^26^, and experimentally infected animals develop only mild inflammation^14^. The low inflammatory tissue reaction, observed in this study, may contribute to the prolonged unapparent infection and difficulty to eradicate the organism, which is typical of canine brucellosis^2,18,20^.

The data generated in this study provided a vast body of novel information that expands our comprehension of canine brucellosis, and provided the basis for new hypothesis regarding transmission of infection to humans or other dogs, through urine, faeces and skin of canine neonates. These hypotheses should be further investigated since it may be extremely valuable to establish new preventive measures when handling neonatal dogs.

In conclusion, *B*. *canis* is widespread in macrophages in foetal and neonatal tissues, and cell tropism is diverse, including enterocytes, renal tubular epithelia, adipocytes, myocardium, and choroid plexus cells. Gastrointestinal infection occurs in congenital brucellosis in dogs. Bacteria may be excreted in the faeces and urine of young puppies.

## Methods

### Experimental design

Canine foetuses and neonates of 19 breeds that died spontaneously in 32 breeding kennels were submitted for necropsy by the owners. Kennels were located in the State of Espirito Santo, Brazil. Puppies were collected from November 2014 until June 2016. Necropsy, sample collection and analysis were performed according to a protocol approved by the Ethics Committee of the Federal University of Minas Gerais (CEUA/UFMG), under protocol number 197/2014. All methods were performed in accordance with the relevant guidelines and regulations.

### *Brucella* diagnosis in puppies - DNA Extraction, PCR and sequencing

For diagnosis of *Brucella-*positive puppies, approximately 50 mg of lung, kidney, spleen, liver, and myocardium, from 200 canine foetuses and neonates were macerated individually with a scalpel blade in 100 μL of TE buffer, and processed for DNA extraction according to the method described by Pitcher *et al*.^41^. After spectrophotometry, DNA samples from the five organs of each puppy were mixed to a final concentration of 250 ng/µL. PCR was directed to the *bcsp*31 gene with primers 5′-TGGCTCGGTTGCCAATATCAA-3′ and 5′-CGCGCTTGCCTTTCAAGGTCTG-3′^42^. PCR reaction was performed with 2.5 μL of sample DNA, 18.5 µL of Supermix PCR (22 mM Tris-HCl [pH 8.4], 55 mM KCl, 1.65 mM Magnesium Chloride, 220 μM dGTP, 220 μM dATP, 220 μM dTTP, 220 μM dCTP, 22 U/mL recombinant Taq DNA Polymerase - Invitrogen, USA), 1.0 µL of each primer (10 µM) in total volume of 25 μL, with expected product of 223 base pairs (bp). Amplification parameters were: 94 °C for 3 minutes; 40 cycles (94 °C for 30 seconds, 60 °C for 30 seconds, 72 °C for 30 seconds) and 72 °C for 10 minutes. The amplified products were subjected to 1.5% agarose gel electrophoresis stained with SYBR®Safe DNA Gel Stain (Invitrogen, USA) and examined in an ultraviolet light transilluminator.

Amplified PCR products of *bcsp31* gene from three different puppies (from different kennels) were sequenced in order to confirm specificity of *Brucella* sp. amplification. Amplicons were extracted from agarose gel and purified using QIAEXII Gel Extraction Kit (Qiagen, USA) and sequenced by capillary electrophoresis in ABI3130 device. Data was analysed as described by Altschul *et al*.^43^ with Phred Software (http://asparagin.cenargen.embrapa.br/phph/), Sequence Scanner Software (Applied Biosystems) (https://products.appliedbiosystems.com/ab/en/US/adirect/ab?cmd = catNavigate2&catID = 600583&tab = DetailInfo) and Blast (https://blast.ncbi.nlm.nih.gov/Blast.cgi).

### Immunohistochemistry

Paraffin embedded tissues from *Brucella-*positive puppies were selected for immunohistochemistry based on the absence of autolysis. The 13 puppies were included in this analysis: four stillborn and nine neonates, from eight different breeds, and nine breeding kennels.

*Brucella* sp. immunolabeling was performed in the following organs: lung, heart, liver, spleen, stomach, intestines, kidney, bladder, prostate, uterus, gonads, umbilical cord, encephalon, and eye. Paraffin embedded tissues were sectioned (4 μm thick) and processed for immunohistochemistry as described by Xavier *et al*.^34^, with modifications. Tissue sections were dewaxed, hydrated, and incubated three times in hydrogen peroxide 10% for 20 minutes, after each bath tissue sections were washed three times in PBS. To block unspecific reactions, tissues sections were incubated in 2.5% powdered milk suspension for one hour. The primary antibody was applied over the slides at the dilution of 1:1000, incubated in humidified chamber overnight and washed in PBS. Secondary biotinylated universal antibody with streptavidin-biotin complex (LSAB2 System-HRP, DAKO, USA) was added and slides were incubated for 1 hour and 40 minutes at room temperature. Reaction was revealed by using the chromogen AEC (DAKO, USA). Meyer’s haematoxylin was used for counterstaining.

The primary polyclonal antibody employed has been previously described^44^. Reactions included positive and negative controls. Positive controls consisted of *Brucella* infected mouse tissues. Negative controls consisted of infected mouse and puppy tissues incubated overnight with PBS with suppression of the primary antibody in the reaction.

Tissue sections were analysed under light microscopy and the frequency of the immunostaining was estimated under high magnification field (400x) as follows: + = one positive mark in ten fields or more; ++ = one positive mark in every two fields; +++ = more than two positive marks in more than ten fields.

### Laser capture microdissection

In order to confirm that immunohistochemically *Brucella* sp.-positive cells indeed contained *Brucella* sp., we performed laser capture microdissection using the MMI CellCut® System. A sample of renal tissue of one stillborn, positive by immunohistochemistry, was used. Paraffin-embedded tissue was mounted on the MMI slide membrane, processed for immunohistochemistry as described, and immunostained tubular epithelial cells were microdissected and subsequently the DNA was extracted using the Dneasy blood & tissue kit (Qiagen, Germany) according to the manufacturer’s instructions. To investigate the integrity of the extracted DNA, polymerase chain reaction (PCR) was performed for the β-actin gene^45^ with 22 μL SuperMix PCR (22 mM Tris-HCl (pH 8.4), 55 mM KCl, 1.65 mM Magnesium Chloride, 220 μM dGTP, 220 μM dATP, 220 μM dTTP, 220 μM dCTP, 22 U/mL recombinant Taq DNA Polymerase (Invitrogen, USA), 0.5 μL of each primer (10 μM), 5′-GGCATCCTGACCCTGAAGTA-3′ and 5′-CGCAGCTCGTTGTAGAAGGT-3′, and 2.5 μL of DNA sample. Amplification was performed at 95 °C for 10 minutes, 95 °C for 30 seconds, 60 °C for 30 seconds, and 72 °C for 30 seconds with 35 consecutive cycles, followed by final extension at 72 °C for 10 minutes, with expected product of 98 bp. The amplified products were subjected to 1.5% agarose gel electrophoresis stained with SYBR®Safe DNA Gel Stain (Invitrogen, USA) and examined in an ultraviolet light transilluminator. For *Brucella* sp. detection, PCR for the *bcsp31* gene was performed as described.

### Isolation and identification of *Brucella canis*

Frozen samples of heart, kidney, spleen and lung, from ten PCR and immunohistochemistry *Brucella-*positive puppies, from eight different kennels, were submitted in duplicates to bacterial isolation^46^. Samples of placenta were available from two puppies, and were included in this study. Tissue samples stored at −80 °C were thawed and homogenized with a scalpel blade in 500 µL of PBS. Then, 100 µL of the homogenate were inoculated in selective Tryptose agar (BD Difco, USA) with and without antibiotics (2,500 UI of polymyxin B; 12,500 UI bacitracin; 50,000 UI of nystatin; 50 mg of cycloheximide; 2.5 mg of nalidixic acid; and 10 mg of vancomycin - Sigma Aldrich, USA) and tryptose broth (BD Difco, USA). Plates were incubated at 37 °C and 5% CO_2_, checked every 48 hours for colony growth. After seven days of incubation, broth was plated on tryptose soy agar without antibiotics and these plates incubated in the same conditions for a maximum of 21 days, when, if no growth was detected, they were considered negative and discarded.

*B*. *canis* isolates were identified based on colony morphology, acriflavine test^47^, PCR for the *bcsp31* gene^42^, and *B*. *ovis*-specific PCR^34^. For molecular analyses, bacterial colonies were collected from agar plates into 200 µL PBS and heat-killed (100 °C/1 hour) and DNA was extracted as described by Pitcher *et al*.^41^.

For the acriflavine agglutination test, 30 μL of a 0.001% aqueous solution of acriflavine was mixed with bacterial colony. PCR for the *bcsp31* gene was performed as described. For *B*. *ovis*-specific PCR 22 μL of SuperMix PCR (22 mM Tris-HCl [pH 8.4], 55 mM KCl, 1.65 mM Magnesium Chloride, 220 μM dGTP, 220 μM dATP, 220 μM dTTP, 220 μM dCTP, and 22 U/mL of recombinant Taq DNA Polymerase) were used (Invitrogen, USA), 0.5 μL of each primer (25 μM), 5′-GCCTACGCTGAAACTTGCTTTTG-3′ and 5′-ATCCCCCCATCACCATAACCGAAG-3′, and 2.0 μL of DNA sample. Amplification was performed at 95 °C for 5 minutes, 95 °C for 1 minute, 57 °C for 1 minute and 72 °C for 1 minute for 35 consecutive cycles, followed by final extension at 72 °C for 5 minutes, with an expected product of 228 bp, which was analysed in 1.5% agarose gel stained with SYBR®Safe DNA Gel Stain (Invitrogen, USA).

Isolates with positive amplification of the *bcsp*31 *Brucella* gene, negative in PCR for *B*. *ovis*, and agglutination in the acriflavine test were identified as *B*. *canis*.

### Data availability

The datasets generated during and/or analysed during the current study are available from the corresponding author on request to anyone who wishes to repeat our analyses or collaborate with us.
